# Pathophysiology and Treatment of Functional Paralysis: Insight from Transcranial Magnetic Stimulation

**DOI:** 10.3390/brainsci13020352

**Published:** 2023-02-18

**Authors:** Giada Pisano, Tommaso Ercoli, Anna Latorre, Lorenzo Rocchi

**Affiliations:** 1 Department of Medical Sciences and Public Health, University of Cagliari, 09124 Cagliari, Italy; 2 Department of Clinical and Movement Neurosciences, UCL Queen Square Institute of Neurology, University College London, London WC1N 3BG, UK

**Keywords:** functional neurological disorders, functional paralysis, transcranial magnetic stimulation, electroencephalography, evoked potentials, cortical inhibition, cortical plasticity, movement disorders

## Abstract

Functional paralysis (FP) or limb weakness is a common presentation of functional movement disorders (FMD), accounting for 18.1% of the clinical manifestations of FMD. The pathophysiology of FP is not known, but imaging studies have identified changes in structural and functional connectivity in multiple brain networks. It has been proposed that noninvasive brain stimulation techniques may be used to understand the pathophysiology of FP and may represent a possible therapeutic option. In this paper, we reviewed transcranial magnetic stimulation studies on functional paralysis, focusing on their pathophysiological and therapeutical implications. Overall, there is general agreement on the integrity of corticospinal pathways in FP, while conflicting results have been found about the net excitability of the primary motor cortex and its excitatory/inhibitory circuitry in resting conditions. The possible involvement of spinal cord circuits remains an under-investigated area. Repetitive transcranial magnetic stimulation appears to have a potential role as a safe and viable option for the treatment of functional paralysis, but more studies are needed to investigate optimal stimulation parameters and clarify its role in the context of other therapeutical options.

## 1. Introduction

Functional paralysis (FP) or limb weakness is a common presentation of functional movement disorders (FMD), accounting for 18.1% of clinical manifestations of FMD [1,2]. According to the largest study in the literature, the mean age at the onset of FP patients is 36.4 years, and women are generally more affected than men (72.9%) [1]. Unilateral symptoms (e.g., hemiparesis or monoparesis) are the most common patterns, but limbs may be affected in any combination [3]. The body distribution may vary during the disease course, especially when other non-motor functional symptoms and psychiatric comorbidities are present [4].

In accordance with Gupta and Lang’s diagnostic criteria, the diagnosis of FP relies mainly on clinical examination, demonstrating incongruence (i.e., clinical features incompatible with known neurological patterns) and/or inconsistency (i.e., variation of patterns over time with susceptibility to distraction) [5,6]. Several positive signs (e.g., Hoover’s sign, hip abductor sign) assessed during the physical examination may guide clinicians to correctly diagnose FP [3,7].

To date, the pathophysiology of FP is not known, and laboratory tests to adequately support clinical diagnosis are lacking [2]. Imaging studies have identified changes in structural and functional connectivity in multiple brain areas, including the supplementary motor area and the temporoparietal junction [8]. However, due to the lack of biomarker studies related to treatment response and prognosis, the relationship between these findings and the pathophysiology of FP is still in its early stages [9].

Evidence from neuroimaging studies suggests that noninvasive brain stimulation techniques may play a key role in the understanding of the pathophysiology of FMD and may represent a possible therapeutic option [10,11]. The aim of this narrative review is to shed light on the role of transcranial magnetic stimulation (TMS) in FP, focusing on the pathophysiological implications and treatment modalities.

## 2. Pathophysiology of Functional Paralysis Investigated by Transcranial Magnetic Stimulation

Previous explanations for FP have proposed that it may be caused by inhibitory influence over the primary motor area (M1) by the orbitofrontal and cingulate cortex, despite physiological activity in premotor areas [12]. Attention seems to be an important factor in the generation of FP since distraction often produces a normalization of symptoms [13], but it does not explain this complex neurological disease alone. In the past two decades, significant advances have been made in understanding the pathophysiology of FP, showing evidence of several deranged neural mechanisms, including those implicated in motor control and preparation [14].

A relevant contribution to the understanding of the pathophysiology of FP has been made by TMS studies. From 2008 to 2020, nine research articles were published on this topic, and their results are reviewed below and summarized in Table 1.

The first paper investigating M1 excitability using TMS in a patient with functional hemiparesis was published in 2008 by Geraldes and colleagues [15], and it showed asymmetric resting motor threshold (RMT) with higher values in the affected hemisphere. Moreover, the motor evoked potential (MEP) recorded from the abductor hallucis of the affected side showed a small amplitude compared with the non-affected one, while central motor conduction time (CMCT) was normal. Other investigations were performed, such as the F wave, which was normal and symmetric. The study was repeated one month after the onset of the symptoms, when the patient was asymptomatic, and showed normalization of the RMT and MEP amplitude. Because of the normal F wave and abnormal M1 excitability on the affected side, the authors proposed that increased inhibitory activation of M1 could be the most likely cause of FP.

In the same year, Liepert and colleagues [16] applied TMS in four female patients with a diagnosis of FP of the left upper extremity. The authors investigated RMT, short-interval intracortical inhibition (SICI), and intracortical facilitation (ICF) at rest. Additionally, single-pulse TMS, at an intensity able to produce MEPs of 1 mV peak-to-peak amplitude at rest, was applied during imagery of tonic adduction of the index finger of the paretic hand, of the unaffected hand, and both simultaneously. The results were compared with an age-matched, but not sex-matched, control group. In all four patients, RMT and CMCT were normal. SICI and ICF values were not significantly different between the two hemispheres in the patients’ group and between patient and control groups, but the former tended to have less ICF. By contrast, MEP amplitude evoked during motor imagery in the affected index finger was significantly lower than that evoked in the non-affected hand and in the control group. According to the authors, the enhanced inhibition observed in FP patients during movement imagination could be seen as the electrophysiological correlate of the patients’ inability to move voluntarily. The same group replicated this result in another eight patients with FP, of which five had “flaccid” paresis and three also had a mixture of fixed dystonia and spasticity [17]. In this study, single-pulse TMS was applied at the intensity of 130% of the individual RMT at rest and during motor imagery. Again, the authors found no relevant differences between patients and controls regarding RMT and MEP amplitudes at rest, but during movement imagination, MEP amplitudes increased by 200% in the control group, decreased by 37% in the paretic hand and increased only by 67% in the unaffected hand in the patient group. These data suggest that the putative inhibitory processes which impair movement in FP are associated with motor planning and are also present when actions are performed by the unaffected side.

The experimental procedure was also extended to lower limb FP during action observation [18]. In this case, ten patients with flaccid paresis and 10 age-matched subjects were studied with a single pulse (115% RMT) and paired-pulse (SICI, ICF) TMS, using a circular coil during imagination of ankle dorsiflexion and while watching another person performing the same movement [19]. At rest, RMT, SICI and ICF were similar in the two groups. During imagery, MEP amplitudes were significantly smaller in patients, confirming the finding of the previous studies, but normal during action observation.

Further exploring the relationship between M1 excitability and motor planning in FP, Morita and coworkers [20] tested MEPs (at a fixed TMS intensity of 80% of the maximal stimulator output) at rest, during tonic contraction (10% of the maximal strength) and in response to an audio cue signal in an FP group of 10 patients, in 9 patients affected by amyotrophic lateral sclerosis (ALS) and 8 healthy controls. Contrary to the other two groups, FP patients did not exhibit the expected latency reduction of MEP during voluntary contraction in both tasks. Additionally, FP patients had larger variability in MEP amplitude in the cued task; both findings possibly reflect variability in the voluntary effort.

Brum and colleagues [21] investigated the extent of contraction-induced facilitation of the MEP, with particular attention to MEP duration, in healthy subjects and patients with paresis of different etiologies, including multiple sclerosis, acute stroke, hereditary spastic paraparesis and FP. They analyzed MEP recorded at rest and during 30% of maximum voluntary contraction at a stimulus intensity of 120% RMT. An increase in MEP duration during muscle contraction was observed in all groups except FP. The authors speculated that this lack of facilitation could be due to the lower excitability of spinal excitatory interneurons.

The largest study on M1 excitability in FP has been performed recently by Benussi and colleagues [22], who measured RMT, SICI and ICF in 21 patients affected by acute onset flaccid FP in the affected and non-affected M1. Findings included increased RMT in the affected M1, compared both to the unaffected side and healthy controls. Moreover, the authors found increased SICI in the affected hemisphere compared to the contralateral one (similar to a previous study by the same group [23]), increased SICI in the affected side and decreased SICI in the unaffected side compared to controls. The increased inhibition in the affected M1 and asymmetry of SICI between the two M1 were proposed by the authors to be disease-specific electrophysiological findings to support the clinical diagnosis of FP.

**Table 1:** Summary of TMS studies investigating the pathophysiology of FP. CMCT: central motor conduction time; ICF: intracortical facilitation; FP: functional paralysis; MEP: motor evoked potential; MSO: maximal stimulator output; RMT: resting motor threshold; SICI: short intracortical inhibition.

Study (Author/Year)	N of Patients Controlled: Yes/No	Stimulation Parameters and Outcome Measures	Findings	Conclusion
Geraldes; 2008 [15]	1	RMT	Asymmetric RMT	Decreased cortical excitability is the most probable cause of FP
	no	CMCT	Small MEP amplitudes in affected abductor hallucis brevis	
			Normal CMCT	
			Normal F waves	
Liepert; 2008 [16]	4 yes	RMT, SICI and ICF at rest	Normal CMCT and RMT	The enhanced inhibition during motor imagery is interpreted as an electrophysiological correlate of the inability to move voluntarily
		MEPs (1 mV) during imagery of tonic adduction of the index finger	SICI and ICF: no difference between the two hemispheres in patients and between patients and healthy controls (but patients had less ICF)	
		CMCT	MEP amplitude in the affected index finger during the motor imagery task was significantly lower compared to not affected hand and control group	
Morita; 2008 [20]	10 yes	MEP (80% MSO), at rest, during tonic contraction (10% of the maximal strength) and in response to an audio cue signal	MEP amplitude increase in the cue signal in ALS and controls Cued-MEP amplitude in FP shows high variability	The increased variability of MEP amplitude could be a supportive parameter for the diagnosis of FP
Liepert; 2009 [17]	8 yes	RMT MEP (130% RMT) at rest and during motor imagery	Normal RMT and MEP amplitudes at 130% RMT at rest During movement imagery MEP amplitudes increased by 200% in healthy controls, decreased by 37% in the FP hand and increased only by 67% in the unaffected hand	Abnormal inhibitory processes in FP (not only of the affected hemisphere) are associated with movement planning and execution
Liepert; 2011 [18]	10 yes	MEP (115% RMT), SICI, ICF during imagination of ankle dorsiflexion and action observation	Normal RMT, SICI and ICF at rest During imagery, MEP amplitudes were smaller in patients but normal during action observation	Abnormally low excitability pattern with down-regulation of motor excitability that might be the electrophysiological substrate of the inability to move voluntarily
Deftereos; 2015 [24]	1 no	MEP (100% MSO) CMCT	Normal MEP and CMCT	Integrity of corticospinal tract
Brum; 2015 [21]	5 yes	MEP (120% RMT) recorded at rest and during 30% of maximum voluntary contraction	Absent increase in MEP duration in the patients with FP during voluntary contraction	The increase in MEP duration during contraction could be due to the contribution of excitatory spinal interneurons to the activation of alpha motoneurons, which is lacking in FP
Jang; 2019 [25]	1 yes	MEP (60% MSO)	Normal MEP	The integrity of the corticospinal tract
Benussi; 2020 [22]	21 yes	RMT, SICI and ICF at rest in the affected and non-affected motor cortex	Increased RMT in the affected M1 compared to the unaffected side and to controls Increased SICI in the affected hemisphere compared to the unaffected side Increased SICI in the affected side and decreased SICI in the unaffected side compared to controls	The asymmetry/imbalance of SICI between the affected and unaffected motor cortex could represent a disease-specific electrophysiological finding

## 3. Therapeutical Transcranial Magnetic Stimulation Studies for Functional Paralysis

In addition to evaluating corticospinal tract integrity and motor cortex excitability, TMS can be used as a therapeutic option in patients with FP. Repetitive transcranial magnetic stimulation (rTMS) consists of short or long, either regular or patterned, consecutive stimuli that can induce long-term changes in cortical excitability by synaptic plasticity, which is assumed to be implicated in its therapeutic effects [26]. RTMS has been applied in several neurological conditions with the intent to improve symptoms, and a few attempts have been made with this purpose also in FP, with nine studies performed in the last 20 years (Table 2).

The first studies were performed on small numbers of patients (single case or few subjects) applying high-frequency rTMS over the motor cortex. Schönfeldt-Lecuona and colleagues [27] delivered 15 Hz rTMS (train length of 2 s and inter-train interval of 4 s, overall yielding 4000 biphasic pulses per day) at an intensity of 110% RMT for the first 2 weeks and 90% RMT for the following weeks, over the M1 contralateral to the affected hand, for a total of 12 consecutive weeks, in a young patient who had suffered from a complete FP of the right arm for 4.5 months. During the second week of treatment, he reported spontaneous jerking of hand muscles for a short period after the stimulation, and he completely recovered at the end of the 12th week. Muscle strength remained normal for the following six months. This protocol was applied by the same authors on four more patients, with variable duration (from 4 to 12 weeks) depending on the timing of recovery [28]. Three patients successfully responded to the treatment, while the 4th dropped out from the study at the 4th week of treatment, without benefit on the symptoms and a further diagnosis of malingering. During the trial, patients received standard therapies (occupational therapy, sports therapy, relaxation techniques, and educational talks) and pharmacological therapy for anxiety/depression, but no cognitive-behavioral, analytical, or otherwise specific psychotherapy. A similar rTMS protocol was then used in a placebo-controlled, single-blind, two-period crossover trial, in which a total of 12 patients with FP were included [29]. Eleven patients were included in the active rTMS condition (consisting of 15 Hz rTMS over the contralateral M1 hand area for 30 min once daily over two periods of five consecutive days at 80% RMT, with a train length of 2 s and an intertrain interval of 4 s). Eight of them also completed the placebo rTMS condition (a real electromagnetic placebo device was placed in front of the stimulation coil; the protocol was otherwise identical to the active condition). After active rTMS, patients showed a significant increase in muscle strength measured with a dynamometer, even in patients that did not receive any other form of treatment; this effect was not observed after sham rTMS.

The study that included the largest number of patients dates back to 2010 [30], in which the authors retrospectively reviewed the medical records of 70 patients with FP who received therapeutic rTMS over nine years. The diagnosis was paraparesis in 57%, monoparesis in 37%, tetraparesis and hemiparesis in 3% each. The rTMS was used for routine diagnostic purposes and consisted of an average of 30 stimuli delivered at low frequency (every 4–5 s) and maximal intensity of 2.5 Tesla with a circular coil over the contralateral motor cortex (or bilaterally for tetraparesis). Another session of 30 stimuli was sometimes delivered a few minutes later in case of incomplete improvement. The treatment was effective in most cases (62 patients, 89%), with a total recovery in 53 patients and a dramatic improvement in nine patients. Patients with acute onset of symptoms had a favorable outcome, whereas this was not the case when psychiatric comorbidity was present.

The specificity of these results was not confirmed in a recent randomized controlled, double-blind, parallel-group trial by the same group, in which patients were randomly assigned to receive either active or sham rTMS for two sessions performed at a 1-day interval [31]. RTMS consisted of 60 consecutive stimuli each day at a frequency of 0.25 Hz, over the motor cortex contralateral to the functional paralysis, bilaterally in the case of paraparesis, and at an intensity above the RMT; a sham coil and identical rTMS protocol was used for the inactive session. A total of 62 patients, 56 with FP, were included in the study, and 32 were randomized in the active group. Interestingly, it was found that two sessions of either active or sham rTMS improved the motor deficit, with no evidence of a difference between the two. This result suggests a placebo effect induced by the rTMS protocol.

Differently from severe lesions of the corticospinal tract, in FP, suprathreshold TMS pulses over M1 can induce movement in the paralyzed limb, showing to the patients that pathways from the brain to the limb are intact and that the weakness is potentially reversible. Based on this notion, McWhirter and colleagues [32] applied a simple rTMS protocol, without additional concomitant treatments, in 10 patients diagnosed with FP affecting one or both upper limbs, in a wide age range (18–75 years). RTMS was administered as 46–70 single pulses (variation reflecting patient preference), in sets of 4–5 pulses 3–4 s apart, at 120–150% of MT, as tolerated and guided by the participant and producing a visible twitch of the hand or arm. Patients were randomized to receive immediate or delayed (after usual care for three months) TMS treatment, which was given together with a pre-defined verbal protocol designed to standardize the effects of suggestion. Overall, there was a significant reduction in self-reported symptom severity immediately after treatment, which was not confirmed by objective measures. According to the authors, many reasons can account for this negative result, including that all the patients had been resistant to previous conventional treatment and that concomitant intensive, multidisciplinary therapy input was not offered. However, the latter explanation is at odds with another similar but retrospective study, in which rTMS was applied in a single session at a supra-threshold intensity to obtain a reproducible EMG response and delivered in sequences of 10 magnetic stimuli (manual trigger) [33]. Among 41 patients recruited, 25 had FP. Symptom resolution was obtained in 78.9% after TMS alone, but the number of FP that was part of this group was not specified. Contrary to the study by McWhirter and coworkers, in which the possibility of the placebo effect was mentioned, in the latter study, patients were told that “TMS is highly efficient to switch on motor circuitry by a complex but as yet unknown action on brain”. Although, in this case, suggestion cannot be excluded, the authors speculated that the evidence of movements induced by stimulation could be a major feedback cue and the key factor explaining the efficiency of TMS.

**Table 2:** Summary of therapeutical TMS studies on FP. CMCT: central motor conduction time; EMG: electromyography; ICF: intracortical facilitation; FP: functional paralysis; M1: primary motor area; MEP: motor evoked potential; MSO: maximal stimulator output; RMT: resting motor threshold; rTMS: repetitive transcranial magnetic stimulation; SICI: short intracortical inhibition; RCT: randomized controlled trial; spTMS: single pulse TMS; TMS: transcranial magnetic stimulation.

Study (Author/Year)	N of Patients Study Design	Stimulation Parameters	Outcome Measures	Follow-Up: Point 1	Follow-Up: Point 2
Jellinek; 1992 [34]	1 Case report	Vertex Single-pulse TMS Figure of eight coil Suprathreshold stimulation	Clinical	Full recovery at 1 week	Sustained recovery (1 month)
Schonfeldt-Lecuona; 2003 [27]	1 Case report	M1 Patterned rTMS at 15 Hz 110% RMT for the first 2 weeks and 90% RMT for the following weeks 4000 pulses per day 12 consecutive weeks	Clinical	Amelioration of distal muscle atrophy and strength, normalization of skin color and limb sensibility	Complete recovery
Schonfeldt-Lecuona; 2006 [28]	4 Case series	M1 Patterned rTMS at 15 Hz 110% RMT for the first 2 weeks and 90% RMT for the following weeks 4000 pulses per day Duration depending on the outcome	6-point rating scale (0–5) of muscle power	¾ improved (1 not FP)	Sustained improvement at 6–12 months
Chastan and Parain; 2014 [30]	70 Retrospective	M1 (unilateral or bilateral) 30 stimuli delivered every 4–5 s, for 2–3 min Maximal intensity of 2.5 Tesla TMS with a circular coil Another session of 30 stimuli was sometimes delivered a few minutes later in cases of incomplete improvement	Clinical	Effective in most cases (62 patients, 89%), with a total recovery in 53 patients and a dramatic improvement in 9	Recurrence in 8; effective in 6 after re-apply
Broersma; 2015 [29]	11 real, 8 sham smastimulation Placebo-controlled, single-blind, two-period crossover trial No other concomitant treatment	M1 15 Hz rTMS (train length of 2 s and an intertrain interval of 4 s) for 20 min once daily 80%RMT 2 weeks (5 days a week)	MRC scale, Dynamometry Subjective improvement	Increase in muscle strength in patients receiving real rTMS	-
McWhirter; 2016 [32]	10 Randomized to immediate (n. 7) and delayed (3 months, n. 3) treatment Verbal protocol designed to standardize the effects of suggestion	spTMS 120–150% RMT, as tolerated and guided by the participant and producing a visible twitch of the hand or arm 4–5 pulses, 3–4 s apart, a total of 46–70 pulses Single session	Measures of disability (SF-12 and MRS) Self-symptom severity (5-point Likert Scale) Hand grip strength (dynamometer) Tapping frequency	Significant improvement in SF-12, MRS and self-symptoms severity report No significant difference in grip strength or tapping frequency	At 3 months follow-up in 8 subjects, no significant differences compared with before treatment in the self-reported symptom severity
Pick; 2020 [35]	21 (10 real, 11 sham) RCT	Real stimulation M1 1 pulse every 5–10 s 120% RMT intensity 2 sessions 4 weeks apart Sham stimulation: Same as real but with 80% RMT intensity	Primary: patient-rated symptom change assessed with the Clinical Global Impression Improvement (CGI-I) Secondary: various clinical scales (see text)	Reported symptom improvement more after the real TMS intervention, with small to moderate effect sizes	Improvement before and/or after 2nd session; 3 months later: sustained improvement
Bonnan; 2021 [33]	25 Retrospective	10 single pulses (circular coil) on each M1 Supra-threshold intensity to obtain a reproducible EMG response Rescue TMS and muscle stimulation	Clinical Global Impression-Improvement (CGI)	High rate of immediate complete recovery after TMS alone	Outcome after one year was poor
Chastan; 2022 [31]	62 (32 real rTMS, 30 sham rTMS) Randomized controlled double-blind parallel-group trial	M1 0.25 Hz RMT intensity 60 consecutive stimuli a day, two sessions performed at a 1-day interval	Clinical examination (motor scores) Primary: success rate defined as a decrease of ≥1 point in the global motor score after rTMS sessions Secondary: fine motor scores and adverse events	13/32 (41%) and 11/30 (37%) patients had increased strength after active or sham rTMS, respectively Changes in both global and fine motor scores were not different between the 2 groups	10/62 were lost to follow-up 24 patients (46%) had a persistent motor deficit, 28 patients had complete improvement, with 22 having received at least one active rTMS session

Lastly, another randomized controlled rTMS trial has been performed recently in FP. In this study by Pick and colleagues (Pick et al., 2020 [35]), patients were randomized to receive active or inactive TMS. The active treatment consisted of 20 single pulse TMS delivered to M1 (corresponding to the participant’s weakest limb), at an interval of 5–10 s and 120% RMT intensity, in two sessions four weeks apart. Before this, patients had received around 100 pulses (every 5–10 s) to find the RMT. The inactive protocol was similar to the active protocol but at 80% RMT. Although this is a “real” TMS protocol, it did not induce any visible hand/foot movement. Patients reported symptom improvement more after the active TMS intervention, with small to moderate effect sizes (the main outcome was subjective); however, some degree of improvement in FND symptoms was observed in both groups prior to commencing the first TMS session, and some patients had additional ongoing interventions.

## 4. Discussion

In general, TMS studies on the pathophysiology of FP are few and present small sample sizes, so results should be interpreted with caution. This should also be stressed in the light of variable control groups, sometimes represented by healthy controls only, others including patients with neurological conditions affecting the corticospinal tract [20,21]. These limitations aside, there is general agreement on the integrity of corticospinal pathways, reflected in the normal CMCT [15,16,24,25]. Conflicting results have been found on the net excitability of M1 in resting conditions, assessed by RMT and MEP amplitude [36]. Whereas the RMT has been reported to be increased in some studies [22,24], this finding has not been confirmed by other authors [16,18], who also found normal MEP amplitudes in FP [17,18]. In this regard, it is worth noticing that a single TMS intensity was used instead of MEP recruitment with multiple stimulation intensities, which may reflect M1 excitability more accurately [37,38,39]. A certain variability in results has also been found in intracortical inhibition processes, with SICI reported to be either normal [16,18] or increased in the affected and decreased in the unaffected hemisphere [22]. If confirmed, this finding might differentiate FP from hyperkinetic functional movement disorders, such as functional dystonia, where reduced SICI has been found [40,41]. Again, it is worth noticing that different intensities of conditioning stimuli were used in the studies and that SICI recruitment is likely to be a better marker of intracortical inhibition due to the variability in SICI threshold across healthy subjects and patients with neurological disorders [38,42,43,44].

A number of findings point towards alterations additional to simple abnormalities of net M1 excitability or circuitry: in particular, one group reported a lower increase of MEP amplitude during motor imagery, both in the affected and non-affected limbs [16,17], possibly reflecting abnormalities in long-range connectivity between M1 and higher-order cortical areas involved in movement planning. This would be in keeping with the finding of decreased activity of cortical areas implicated in motor preparation, such as the supplementary and pre-supplementary motor areas, observed in functional neuroimaging studies [14,45,46].

Lastly, the possible involvement of spinal cord circuits remains an underinvestigated area, with a single study reporting normal motoneuronal excitability [15] and another hypothesizing lower excitability of local excitatory interneurons [21].

Therapeutic TMS studies have been performed with variable protocols and yielded mixed results. In some reports, rTMS was applied with a high frequency, albeit with a slightly different stimulation intensity, with the aim of inducing cortical plasticity [27,28]; results were generally favourable, with improvement in patients’ strength. It is worth noticing, however, that only one of these studies was controlled [29]; therefore, a placebo effect of rTMS cannot be excluded. This notion is further supported by other findings, such as patients’ improvement with very low stimulation frequency, outside the range of plasticity-inducing rTMS protocols [30,35], in addition to strength increase with sham stimulation [31]. Along this line, it has been suggested that possible therapeutic effects of TMS on FP may rely on the demonstration of intact motor pathways to the patient in an objective way [33,34]. Further issues which have not been sufficiently considered include the lack of a clear rationale for the choice of the cortical area to stimulate and the specificity of the TMS effect with respect to other therapies. The latter is a crucial factor that has seldom been addressed. Only a few studies clearly state that the beneficial effects of TMS were observed independently from other forms of therapy [29] or that patients’ improvement might have been due to other ongoing treatments [27,28].

In conclusion, rTMS appears to have a potential role as a safe and viable option for the treatment of FP; however, more studies are needed to investigate optimal stimulation parameters and clarify its role in the context of other therapeutical options.
